# Prognostic impact of mismatch repair genes germline defects in colorectal cancer patients: are all mutations equal?

**DOI:** 10.18632/oncotarget.5395

**Published:** 2015-10-15

**Authors:** Elena Maccaroni, Raffaella Bracci, Riccardo Giampieri, Francesca Bianchi, Laura Belvederesi, Cristiana Brugiati, Silvia Pagliaretta, Michela Del Prete, Mario Scartozzi, Stefano Cascinu

**Affiliations:** ^1^ Clinica di Oncologia Medica e Centro Regionale di Genetica Oncologica, Università Politecnica delle Marche, Azienda Ospedaliero-Universitaria Ospedali Riuniti, Ancona, Italy; ^2^ Medical Oncology, Azienda Ospedaliero-Universitaria di Cagliari, Monserrato (CA), Cagliari, Italy

**Keywords:** Lynch syndrome, germline mutation, colorectal cancer, prognosis, genetic testing

## Abstract

**Background:**

Lynch syndrome (LS) is the most common hereditary colorectal cancer (CRC) syndrome, caused by germline mutations in MisMatch Repair (MMR) genes, particularly in MLH1, MSH2 and MSH6. Patients with LS seem to have a more favourable prognosis than those with sporadic CRC, although the prognostic impact of different mutation types is unknown.

Aim of our study is to compare survival outcomes of different types of MMR mutations in patients with LS-related CRC.

**Methods:**

302 CRC patients were prospectively selected on the basis of Amsterdam or Revised Bethesda criteria to undergo genetic testing: direct sequencing of DNA and MLPA were used to examine the entire MLH1, MSH2 and MSH6 coding sequence.

Patients were classified as mutation-positive or negative according to the genetic testing result.

**Results:**

A deleterious MMR mutation was found in 38/302 patients. Median overall survival (OS) was significantly higher in mutation-positive vs mutation-negative patients (102.6 vs 77.7 months, HR:0.63, 95%CI:0.46–0.89, *p* = 0.0083). Different types of mutation were significantly related with OS: missense or splicing-site mutations were associated with better OS compared with rearrangement, frameshift or non-sense mutations (132.5 vs 82.5 months, HR:0.46, 95%CI:0.16–0.82, *p* = 0.0153).

**Conclusions:**

Our study confirms improved OS for LS-patients compared with mutation-negative CRC patients. In addition, not all mutations could be considered equal: the better prognosis in CRC patients with MMR pathogenic missense or splicing site mutation could be due to different functional activity of the encoded MMR protein. This matter should be investigated by use of functional assays in the future.

## INTRODUCTION

Lynch syndrome (also known as Hereditary Non Polyposis Colorectal Cancer, HNPCC), is an autosomal dominant disorder accounting for 3–5% of all CRC cancer cases [1], representing the most common hereditary colorectal cancer (CRC) syndrome.

Lynch syndrome is caused by germline mutations in any of the MisMatch Repair (MMR) genes, even if mutations in MLH1 (located on 3p21), MSH2 (on 2p22-p21) and MSH6 (2p16) account for almost 90% of identified defects [2]. These genes are responsible for repair of DNA mismatch errors, arising during DNA replication as a result of either incorrect base pairing or slippage of DNA polymerization on the template strand. Failure of DNA mismatch repair leads to a phenomenon defined microsatellite instability (MSI), characterized by the change of the length of simple, repetitive nucleotide sequences that occur throughout the genome.

Lynch syndrome predisposes mutation carriers to colorectal cancer, with a lifetime risk about 80%, as well as to other extracolonic malignancies, including carcinomas of the endometrium, ovary, small bowel, stomach, ureter, biliary tract, pancreas, prostate and some different types of skin cancers [3, 4].

Genetic testing for Lynch syndrome has evolved as a powerful clinical tool: the detection of a pathogenic MMR mutation in the proband enables the identification of other mutation carriers into the same family who would benefit from risk-reduction strategies, such as cancer surveillance and prophylactic surgery. However, mutation testing can provide three categories of possible results: pathogenic mutation, variant of uncertain significance (VUS), or informative negative finding, if no mutations are found [5].

Pathogenic MMR-genes mutations can involve any part of each MMR genes, in the absence of mutational “hot spots”. Their nature is also extremely variable: frameshift, non-sense, splicing-site, missense mutations and large gene rearrangements could all be responsible of defective MMR system.

Lynch Syndrome-related CRC are characterized by typical histopathologic characteristics, such as poor differentiation, increased proportion of mucinous and signet-ring cell carcinomas, lymphocytic infiltration and the presence of MSI in more than 80% of these cases. An accelerated progression from adenoma to colorectal adenocarcinoma also occurs [6, 7].

The knowledge of this syndrome in practice may be useful also from a prognostic point of view: it is suggested that Lynch Syndrome associated CRC might have a better prognosis than sporadic CRC [8–10].

However, the prognostic impact of different types of mutation (such as truncating, splice-site, missense and large gene rearrangement mutations) is still unknown.

The aim of our study is to compare survival outcomes of different types of MMR-genes mutations in patients with Lynch Syndrome-related CRC.

## RESULTS

Between July 1996 and March 2014, 302 consecutive colorectal cancer patients were enrolled into this study. All patients met at least one of Bethesda guidelines criteria, while 84 (27.8%) fulfilled Amsterdam criteria.

Median age at diagnosis was 49 years (range 18–85 years). Male/female ratio was 50%/50%.

Patients' characteristics are summarized in Table 1.

**Table 1:** Clinical and pathological characteristics of patients according to mutational status

Patients characteristics			
**Number of patients**		**302**	
**Sex**			
• **Male**		151 (50%)	
• **Female**		151 (50%) 151 (50%)	
**Median age (range)**		49 (18–85)	
**Patients fulfilling Bethesda Guidelines**		302 (100%)	
**Patients fulfilling Amsterdam criteria**		84 (27.8%)	
**Clinical and pathological characteristics^a^**			
	**Mutation positive *n* = 38**	**Mutation negative *n* = 252**	***P* value**
**Gender**		
• Males	22 (57.9%)	126 (50%)	0.32
• Females	16 (42.1%)	126 (50%)	
**Age**		
• <50	26 (68.4%)	128 (50.8%)	0.06
• ≥50	12 (31.6%)	124 (49.2%)	
**Site**			
• Right colon	20 (52.6%)	62 (24.6%)	**0.0002**
• Left colon	5 (13.2%)	97 (38.5%)	
• Rectum	4 (10.5%)	40 (15.9%)	
• Multiple colorectal tumours	6 (15.8%)	11 (4.4%)	
• Not reported	3 (7.9%)	42 (16.6%)	
**Multiple *vs* Single colorectal tumours**			
• Single	29 (76.3%)	199 (79%)	**0.0110**
• Multiple	6(15.8%)	11(4.4%)	
• Not reported	3(7.9%)	42(16.6%)	
**Stage (TNM)**			
• I	11 (28.9%)	52 (20.6%)	0.38
• II	12 (31.6%)	56 (22.2%)	
• III	9 (23.7%)	75 (29.8%)	
• IV	1 (2.6%)	34 (13.5%)	
• Not reported	5 (13.2%)	35 (13.9%)	
**Grade**			
• G1	4 (10.5%)	38 (15.1%)	**0.04**
• G2	14 (36.8%)	107 (42.5%)	
• G3	11 (28.9%)	31 (12.3%)	
• Not reported	9 (23.7%)	76 (30.1%)	
**Histopathological characteristics**			
• Mucinous or signet-ring cell component	18 (47.4%)	37 (14.7%)	**< 0.0001**
**Presence of synchronous, metachronous colorectal or other HNPCC-associated tumours**	19 (50%)	59 (23.4%)	**0.0012**

a Patients with VUS (*n* = 12) are not shown in the table because they were excluded from the analysis

Sex, age and stage were not significantly different between mutation-positive and mutation negative patients whereas a significant association between mutational status and CRC location was observed (*p* = 0.0002): mutation-positive cases were more frequently right-sided (52.6%) than mutation-negative patients (24.6%). G3 tumours were more frequent in in mutation-positive subjects (28.9% *vs* 12.3% *p* = 0.04). The presence of a mucinous or signet-ring cell component was also more frequent in mutation-positive patients (47.4% *vs* 14.7% *p* = < 0.0001). Moreover, the presence at diagnosis of multiple synchronous colorectal malignancies, metachronous colorectal cancers or other HNPCC-associated tumours resulted more frequent among mutation-positive patients than mutation negative cases (50% *vs* 23.4%, *p* = 0.0012) (Table 1).

### MSI analysis

Tumour samples adequate for MSI assessment were available only for 78 patients. We were unable to obtain tumour samples for MSI analysis from the remaining patients because they underwent surgery in other hospitals.

MSI-H was found in 22 patients (28.2%), among them 13 harboured a MMR genes pathogenic mutation; 14 patients (18%) had MSI-L tumour and in 42 cases (53.8%) MSS was observed. None of MSI-L or MSS patient was carrier of a MMR pathogenic mutation.

The CAT25 microsatellite analysis showed instability in all 22 patients with MSI-H.

### Immunohistochemical analysis (IHC)

Slides for IHC analysis of MLH1 and MSH2 expression were available for 89 patients. Loss of MLH1 expression was detected in 15 out of 89 cases (16.9%), loss of MSH2 expression was observed in 10 patients (11.2%) and loss of MSH6 expression was found in 18 cases (20.2%).

### MLH1, MSH2 and MSH6 genes mutations

All 302 patients underwent genetic testing using direct DNA sequencing. Cases who tested negative for the mutational analysis were investigated by MLPA analysis.

Globally, 43 different mutations in 65 patients were found, while in 237 patients the test resulted negative.

We found 26 patients with MLH1 gene mutations: 1 patient had a large rearrangement, 8 patients harboured splice-site mutations, 14 patients had a missense mutation and 3 patients had a silent mutation. There was a high heterogeneity of mutation types, with 17 different mutations identified (1 large rearrangement, 4 splice-site, 9 missense end 3 silent-mutation).

We identified 35 patients harbouring MSH2 gene mutations: 6 patients had a large rearrangement, 4 patients harboured a frameshift, 3 patients had a non-sense mutation, 18 patients carried a missense mutation, 2 patients a silent mutation and other 2 an intronic variant. Even in this group of MSH2-mutated patients heterogeneity of mutation types was observed, with 23 different mutations types (3 large rearrangements, 4 frameshift mutations, 2 non-sense mutations, 10 missense, 2 silent-mutations and 2 intronic variants) discovered.

In the MSH6 gene 4 different mutations were found in 4 patients: 1 frameshift, 1 missense mutation and two intronic variants.

Among mutation carriers, we identified 38 patients who carried a definitely pathogenic or likely pathogenic mutation (Table 2), 15 patients who carried a not pathogenic or a likely not pathogenic mutation and 12 patients who carried a Variant of Uncertain Significance (VUS): this latter group of patients, as previously stated, was excluded from further analysis.

**Table 2:** CRC patients carriers of a MMR-genes pathogenic mutation

Patients with pathogenic MMR-genes mutations					
Number of carrier patients	Type of mutation	Mutation gene and site	IARC Class	INSIGHT	Functional assays (26,32)
1	Large rearrangement	MLH1 del 16–19			
2	Large rearrangement	MSH2 del EX 7–8			
1	Large rearrangement	MSH2 del EX 1–2			
3	Large rearrangement	MSH2 del EX 1–7			
2	Splice acceptor site	MLH1: intron 13 IVS13c. 1559–1G > T	5	Reported	
1	Splice acceptor site	MLH1: intron 3 (IVS3-A > G) (307–2)		Not reported	
1	Splice donorsite	MLH1: ex13 VS13+1G > T (1558+1G > T)	5	Reported	
4	Splice donorsite	MLH1: intron 9 IV9 c.790 +4A > T		Not Reported	Pathogenic (36)
1	Frameshift	MSH2:ex15 c.2629delAG E876fsX879	5	Reported	Pathogenic
1	Frameshift	MSH2: ex2 Frameshift 93 (del TT al 278)	5	Reported	
1	Frameshift	MSH2:ex3 (del GA al 611) Stop 231		Not Reported	
1	Frameshift	MSH2:ex13 c.2145delT stop719		Not Reported	
1	Frameshift	MSH6:ex4 c.1815delTA T605fsX638		Not Reported	
1	Non-Sense	MSH2:ex12 R621X (1861 C > T)	5	Reported	
2	Non-Sense	MSH2:ex3 Q170X (508 C > T)	5	Reported	
1	Missense	MLH1:ex17 P648S (1942 C-T)	5	Reported	
1	Missense	MLH1:ex18 R687W (2059 C > T)	5	Reported	
1	Missense	MLH1:ex9 N260L (779 T > G)	5	Reported	
1	Missense	MLH1: ex4 T117M (350 C > T)	5	Reported	
1	Missense	MLH1: ex15 L559R (1676 T > G)	4	Reported	
7	Missense	MSH2: ex7 R359S (1077 A > T)	5	Reported	Pathogenic
1	Missense	MSH2: ex3 G162R (484 G> C)		Not reported	Pathogenic
2	Missense	MSH2: ex3 V161D (482 T > A)	3	Reported	Pathogenic

Out of 38 patients who carried pathogenic MMR-genes mutations, in 31 patients the mutations were found using direct sequencing while in 7 patients the mutations were detected using MLPA.

Eighty-four patients (27.8%) fulfilled Amsterdam criteria and the mutation rate in this group of patients was 33%: 28/84 patients were found to have a pathogenic mutation, 1/84 resulted carrier of a VUS, while the remaining 57/84 patients resulted negative to mutation testing.

As expected, the detection rate resulted to be higher in patients fulfilling Amsterdam criteria than patients fulfilling Bethesda Guidelines (33.3% *vs* 12.6%).

### Survival analysis

On the basis of genetic testing results, patients were classified in 2 aforementioned groups:

- 38 *mutation-positive* patients
- 252 *mutation-negative* patients (comprising 237 patients whose test was negative and 15 patients in which a not pathogenic variant was found)

Median follow-up time was 79 months (range 4–487). Median overall survival (OS) for all patients was 79.4 months.

**Figure 1:**
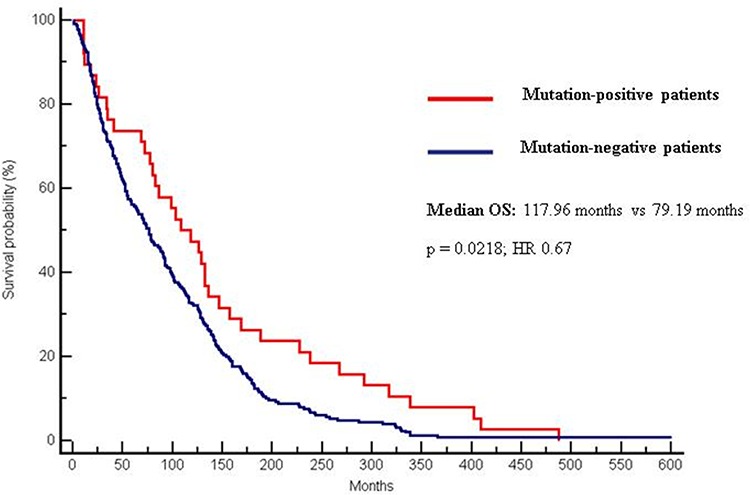
Differences in OS between mutation-positive (117.96 months) and mutation negative patients (79.19 months). (*p* = 0.0218; CI 0.51–0.94; HR 0.67)

Median OS was significantly higher in the mutation-positive group (117.96 months) compared to the mutation-negative group (79.19 months) (*p* = 0.0218; CI 0.51–0.94; HR 0.67) (Figure 1).

**Figure 2:**
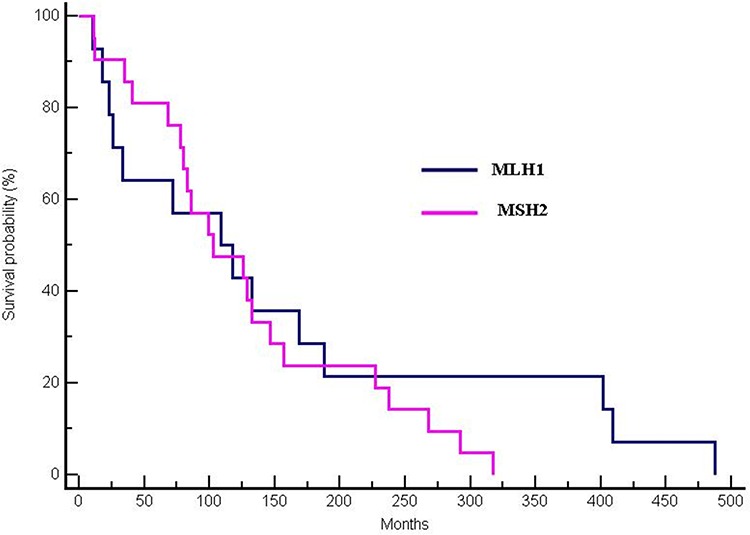
Differences in OS between MLH1 and MSH2-mutated patients (median OS 117.96 months for MLH1-mutated vs 102.62 months for MSH2-mutated patients, *p* = 0.42; HR:0.77; 95% CI 0.36–1.52)

In 38 mutation-positive patients, no survival differences were observed between MLH1 and MSH2-mutated patients (median OS 117.96 months for MLH1-mutated *vs* 102.62 months for MSH2-mutated patients, *p* = 0.42; HR:0.77; 95% CI 0.36–1.52) (Figure 2).

**Figure 3:**
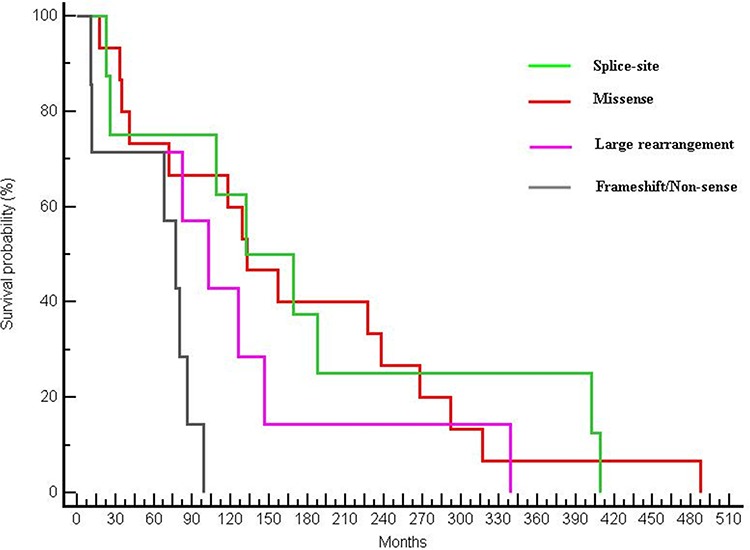
Differences in OS between patients with different types of mutations (OS of 132.46 months in patients with a missense mutation, 150.46 months for patients with a splice-site mutation, 102.62 months for patients with a large rearrangement and 77.31 months in patients with a frameshift or non-sense mutation; *p* = 0.0224)

Looking at different types of mutation, a significant difference in terms of OS was found: median OS was 132.46 months for patients harbouring a pathogenic missense mutation, 150.46 months for patients with a splice-site mutation, 102.62 months for patients with a large rearrangement mutation and 77.31 months in patients in which a truncating mutation (frameshift or non-sense) was found (*p* = 0.0224) (Figure 3).

**Figure 4:**
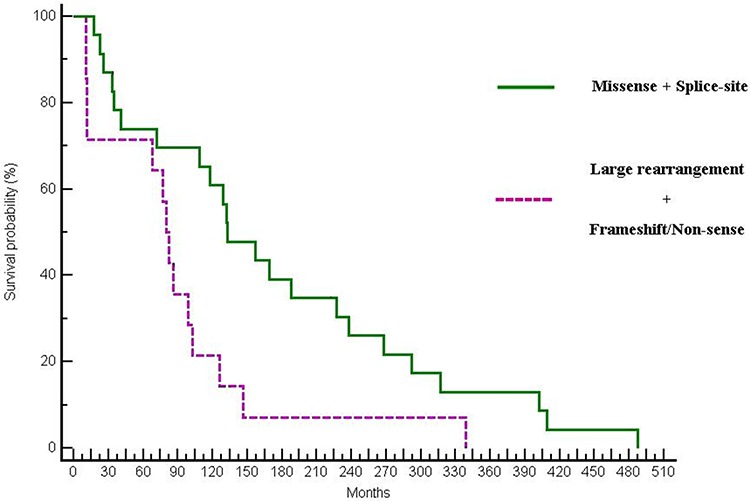
Differences in OS between patients with missense and splice-site mutations vs patients harbouring large rearrangement or truncating mutations (132.46 vs 82.55 months; *p* = 0.0153; HR:0.46; 95% CI:0.16–0.82)

Finally, as in the primary objective of this analysis, when looking at the differences in OS between mutations with a favourable outcome (such as missense and splice-site mutations) compared with mutations with a likely more impaired gene expression such as large rearrangements, nonsense or truncating mutations, a statistically significant difference in OS was observed (132.46 vs 82.55 months; *p* = 0.0153; HR:0.46; 95% CI 0.16–0.82) (Figure 4).

### Multivariate analysis

At multivariate Cox regression analysis, the presence of missense and splice-site mutation maintained its prognostic role (*p* = 0.0011).

On the other hand, among other analyzed variables, only stage resulted independently associated with improved OS, while the presence of mucinous or signet-ring cell histology was associated with a poorer prognosis.

## DISCUSSION

Given the significant differences between the biology of MMR-deficient and -proficient colorectal cancers, the prognostic role of MMR-genes mutations in CRC patients has been widely investigated in several clinical series and reviews, suggesting that patients with MSI CRC and Lynch Syndrome could have a better prognosis than patients with sporadic CRC [8, 9, 11].

However, our study represents the first analysis in order to verify the prognostic impact of different mutations in patients with HNPCC.

Our findings confirm the favourable prognostic impact of MMR-genes mutations [10, 12, 13].

At present, there is no clear explanation for the survival advantage of Lynch Syndrome CRC patients. Among several proposed hypotheses, increased immunogenicity of MSI-H CRC is one of the most discussed, as suggested by the frequent presence of activated intraepithelial T-lymphocytes. Immune activity could be explained due to the accumulation of mutations in genes encoding cell surface proteins caused by MMR-system deficiency [14–16]. Further molecular evidence for the immunogenicity of MSI-H colon cancers has been provided by gene expression studies demonstrating an up-regulation of several genes associated with an enhanced immune response [17]. In addition, recent data seem to suggest that both sporadic MSI-H and Lynch Syndrome-related colorectal cancer could share some therapeutically important biomarkers, such as significantly higher Thymidylate Synthase (TS) expression (potentially explaining the supposed reduced clinical benefit from 5-Fluorouracil), higher Programmed cell death-1 (PD-1)-positive tumor infiltrating lymphocytes (TIL), BRCA1/2 and CTNNB1 mutations. The sum of these factors might suggest MSI-H as a more promising group for targeted immunotherapy, PARP and Wnt pathway inhibitors [18].

Unfortunately, in Our study the number of patients who were found having TILs at the histological report was rather small. In addition to that, due to the lack of histological tissue from all patients (most patients underwent surgical resection or biopsy in another Centre) it would be impossible to evaluate “de novo” TILs expression and in particular the differences among the various lymphocytes populations.

Moreover, MSI-positive CRC seem to show a reduced microvessel density, due to a lower vascular endothelial growth factor (VEGF) levels [19–21] and, consequently, a reduced metastatic potential. Another explanation could be that MMR defects can affect the viability of tumour cells through the accumulation of mutations in genes necessary for cancer cell survival. To support this hypothesis the evidence showed that MSI-positive CRC are less likely to carry mutations of TP53, KRAS and DCC genes, which are usually associated with uncontrolled cell proliferation and poor prognosis [22–24].

Our study is the first to show a different outcome for patients with different MMR mutations: indeed, our study suggests that overall survival for the group of patients with a less favourable mutation profile is rather poor (10-year survival rate of about 20%). If we compare this result with the10-year survival rate of mutation-negative patients included in the analysis (about 30%) it is easy to refuse the claim that all MMR mutations have the same prognostic impact. Indeed, survival advantage remained remarkable only for subject with a MMR pathogenic missense and splicing site mutation.

The reasons underlying this prognostic difference among patients harbouring different MMR germline mutations remain still unknown, but could be due to the type of functional defect, and therefore, in a different functional activity of the encoded MMR protein.

In fact, while the pathogenic role of some mutations, such as frameshift and non-sense, is clearly predictable because their product consists in a truncated protein, for patients with a missense mutation, the molecular mechanism underlying abnormalities of MMR function is more complex and still to be fully clarified: it may be linked both to the effects that a missense mutation cause on the encoded protein (such as functional and structural alterations related to chemical and physical properties of the involved aminoacid), or to the mutation-affected site of the protein.

The identification of the “clinical relevance” of the mutation that has been identified will be even more crucial if we consider that, with the introduction of next-generation-sequencing methods, a greater number of mutations of unknown clinical significance will be identified. In particular, recent published works point out at other genes implied in colorectal cancer pathogenesis (APC, MUTYH, STK11 and BRCA1/2) as explanations of familial colorectal cancer syndromes in patients who do not present mutations in standard MMR protein gene system [25].

In this setting, *in silico* and *in vitro* functional assays may be used in order to extensively comprehend the defect that each type of mutation causes on the corresponding protein and to estimate its residual functionality and, thus, to verify a possible correlation between the genotype (represented by different types of mutation) and the patient phenotype.

However, at the moment, functional assays are mostly employed to classify VUS, but soon they also could obtain a wide application in clinical practice as suggested by our previous work [26].

Another potential limitation of our work is the definition of patients who are considered “mutation-negative”. Indeed, while our analyses prove that this group of patients does not harbour mutations in hMLH1, hMSH2, hMSH6 genes, we do not know if those patients harbour other (less frequently represented) mutations. For example, PMS2 mutations are nowadays considered as other potential mechanisms of familial colorectal cancer syndromes akin to the more common Lynch Syndrome. Our laboratory is unable to perform PMS2 mutations analysis, due to the technical difficulties in analysing PMS2 gene as a result of a large number of pseudogenes has possibly lead to underreporting of PMS2 mutations in patients with LS. In addition, IHC for PMS2 could not be performed due to the lack of tumour tissue for all patients eligible for analysis. Accounting for the relatively small frequence of PMS2 mutations (up to 8% of all Lynch syndromes) there is a relatively small probability that it would influence the results of our work [27].

In conclusions, our study suggests that, even if MMR-genes pathogenic mutations are generally associated with an improved overall survival in patients with CRC, not all the mutations could be considered equal: their prognostic impact may differ on the basis of the type of mutation and the better prognosis in CRC patients harbouring a MMR pathogenic missense or splicing site mutation could be due to a different functional activity of the encoded MMR protein.

In the future, further multicentric studies enrolling a larger number of patients, in addition to the use of functional assays, could be useful to further explore our insights on the matter.

## MATERIALS AND METHODS

### Patients

The present study included consecutive patients with histologically proven colorectal cancer who underwent genetic testing for Lynch Syndrome at the Centro Regionale di Genetica Oncologica, AO Ospedali Riuniti-Università Politecnica delle Marche-Ancona.

Patients who met Amsterdam criteria I and II [28, 29] or the Bethesda Guidelines [30] were selected to perform genetic testing.

All participating patients were informed of the aim of the study before study entry and signed a written informed consent.

This study was approved by the local ethical committee.

### Clinical and pathological data

Personal and familial cancer history, including site and type of cancer as well as age at diagnosis, was collected from the proband. Pedigrees were traced back for at least three generations and laterally to second- and third-degree relatives.

Cancer diagnoses and deaths in relatives were confirmed by medical or pathologic records.

Histological report provided information about tumour location and extension in the bowel wall, adjacent organs and lymph nodes and other, tumour grade and other histopathological characteristics.

### Microsatellite instability (MSI)

Colorectal cancer DNA was investigated for MSI using the 5-markers panel (two mononucleotide repeats – BAT25 and BAT26- and three dinucleotide repeats –D2S123, D5S346, D17S250) recommended by international guidelines [31].

Microsatellite sequences were PCR amplified from tumour and matched normal DNA using 5′- fluorochrome labelled oligonucleotide primer pairs [32]. PCR products were analyzed by capillary gel electrophoresis (ABI 310 Genetic Analyzer-Applied Biosystems) followed by automated allele sizing using the GeneScan 3.7 software (Applied Biosystems, Foster City, CA). PCR primers and conditions are available from the corresponding Author. Tumours were classified as highly unstable (MSI-H) when at least two of 5 markers were positive, or if instability was found in at least 40% of the analyzed microsatellite markers [33]. CAT25 microsatellite was also studied in all patients [34].

### Immunohistochemical analysis (IHC)

Immunohistochemical (IHC) analysis of MLH1, MSH2 and MSH6 protein expression was performed on 2 μm sections of paraffin-embedded tumour tissue samples, following antigen retrieval [35]. The following primary antibodies and dilutions were used: anti-MLH1 protein (clone G168-728, PharMingen, San Diego, CA), 1:50 dilution; anti-MSH2 protein (clone FE11, Calbiochem/Oncogene Research Products, Cambridge, MA), 1:100 dilution; anti-MSH6 protein (clone H-141, Santa Cruz Biotechnology, Santa Cruz, CA) 1:250 dilution [36].

### Mutational analysis

Mutational analysis was performed on genomic DNA purified from peripheral mononuclear blood cells, using the “Flexigene 3 ml Blood kit (from Qiagen, Germany) according to the instructions of the manufacturer.

All the MLH1, MSH2 and MSH6 exons, including flanking intronic regions, were individually amplified and directly sequenced using “Genetic Analyser 310” and/or “Dx 3500 Genetic Analyser” automated sequencers as previously described [33].

Identified mutations were confirmed on a second sample PCR product. Primer sequences are available from the corresponding Author upon request.

Our results were compared with MLH1, MSH2 and MSH6 normal sequence (respectively available at HNPCC database, http://www.insight-group.org).

### Multiplex ligation-dependent probe amplification (MLPA) analysis

Cases who tested negative for the mutational analyses were investigated for the presence of genomic rearrangements, including the deletion or duplication of one of more exons in the MLH1, MSH2 and MSH6 genes by Multiplex Ligation-dependent Probe Amplification (MLPA) analysis. MLPA was performed with 200 ng of normal and tumour DNAs using the MRC Holland (Amsterdam, Holland) HNPCC probe kit, according to the supplier's protocol. One microliter of the FAM labelled PCR product was then mixed with 1 (l of fluorescent GeneScan 500 LIZ size standard (Applera) in 15 (l of HiDi Formamide, run on an automatic ABI310 DNA analyzer, and evaluated with GeneScan sofware (Applera). The electropherograms showed specific peaks corresponding to each exon of MSH2 and MLH1, as well as additional peaks corresponding to control sequences mapping on different chromosomes. A 40–55% decrease of the area of an MLH1, MSH2, MSH6 exon peak compared to the wild-type control samples was considered as indicative of a heterozygous deletion of that exon.

### Mutational classification

Interpretation of identified mutations' pathogenic significance was performed searching each variant in the InSiGHT (available at http://www.insight-group.org) and then mutations were classified according to the five-class system IARC system recommended by the International Agency for Research on Cancer (IARC) [5].

### Patients classification according to mutational status

Analyzed patients were classified in two categories:

- ***Mutation-positive***: if they were carriers of a definitely pathogenic or likely pathogenic mutation
- ***Mutation-negative***: if genetic testing resulted negative or if they were carriers of a not pathogenic (or of no clinical significance) mutation or of a likely not pathogenic or of little clinical significance mutation

Patients who carried a Variant of Uncertain Significance were excluded from the analysis, due to the uncertain role of this class of mutations.

### Statistical analysis

The design of our study is based on the cohort study model. We assumed that exposed patients are those who harbour mutations of MMR genes with high likelihood of lacking gene function such as frameshift, nonsense mutations and large genetic rearrangements, whereas control patients are those who harbour mutations of MMR genes with more favourable outcome such as splice-site alterations and missense mutations.

On this basis, to test the hypothesis that exposed patients have poorer overall survival compared with the control group, assuming that 10-year overall survival rate in the control group is 70% compared with 20% of the exposed group, with one-sided alpha test level at 0.05 and a statistical power of the test of 0.90, at least 30 patients (15 in each group) are necessary to test this hypothesis.

Statistical analysis was performed with the MedCalc package (MedCalc Statistical Software version 14.10.2 (MedCalc Software bvba, Ostend, Belgium; http://www.medcalc.org; 2014).

Tested variables included: age (<50 *vs*. ≥ 50 years), gender (female *vs* male), tumour location (right *vs* left/rectal tumours), multiple *vs* single colorectal tumours, stage, grade, presence of mucinous or signet-ring cell component, presence of synchronous or metachronous CRC or other HNPCC-associated tumours.

The association between categorical variables was estimated by Chi-square test.

Survival distribution was estimated by the Kaplan–Meier method. Significant differences in probability of surviving between the strata were evaluated by log-rank test. Overall survival (OS) was defined as the interval between the diagnosis to death or last follow-up visit.

Multivariate analysis was performed using Cox multiple regression model including only those variables that resulted significantly related with different outcome at univariate analysis.
